# Operationalizing Surveillance as a Malaria Intervention: Data Use for Action

**DOI:** 10.4269/ajtmh.22-0153

**Published:** 2022-04-25

**Authors:** Arantxa Roca-Feltrer, Baltazar Candrinho, Médoune Ndiop, Debra Prosnitz, Molly Robertson, Yazoume Ye

**Affiliations:** ^1^ Malaria Consortium, United Kingdom;; ^2^ National Malaria Control Programme, Mozambique;; ^3^ National Malaria Control Program, Senegal;; ^4^ PMI Measure Malaria Project, ICF, Rockville, MD;; ^5^ PATH, Seattle, WA

Public health surveillance is an essential pillar of public health, and it is traditionally referred to as the systematic and ongoing collection, collation, and analysis of health-related data for timely decision making to address programmatic gaps and optimize resource allocation.^1^^,^^2^ In 2015, the WHO Global Technical Strategy for Malaria 2016–2030 emphasized surveillance as a core intervention for accelerating progress toward malaria elimination across endemic settings. The WHO further emphasized the importance of surveillance in the revised May 2021 Global Technical Strategy.^3^^,^^4^ However, although significant efforts have been made, particularly in the malaria elimination context, in defining the requirements and core principles of a functional surveillance system, the focus has been placed on generating and integrating high-quality data, whereas little attention has been given to data use and data-to-action components. The full potential of the operationalization of malaria surveillance as a core intervention across the transmission continuum is yet to be realized in many medium- to high-transmission settings. Upgrading malaria surveillance as a core intervention implies that a fit-for-purpose system is in place, as well as the effective use of its generated data for decision making. Weak malaria surveillance systems result in national malaria control programs lacking up-to-date, high-quality data, and, therefore, they are not always able to implement and monitor effectively and efficiently appropriate public health interventions. A robust surveillance data collection system and a framework for using these data for action need to be both fully operational and in sync to achieve the desired impact.

To articulate timely data-informed actions in routine programs effectively, it is necessary to sync and optimize the use of routine malaria information reported through different systems, such as those generated by outpatient departments, antenatal care programs, community health workers, and the private sector; adapt the granularity of the data to local needs (e.g., district or large city versus regional or national); invest in effective data-to-action frameworks through clear governance and coordination mechanisms; and promote adaptive surveillance to respond to evolving global threats (e.g., the COVID-19 pandemic) and changing external factors. The articles in this *Journal *series of supplements provide key recommendations based on the lessons learned from operationalizing a range of different surveillance components across a wide range of geographies and transmission settings. This supplement also delves into how malaria surveillance is effectively being transformed into a core intervention, describes examples of how routine data can be used to guide programming of malaria interventions across varying transmission settings, and details its critical importance in the “COVID-endemic” context. Articles in the *Journal *supplement review malaria surveillance system components, provide a conceptual framework for effective surveillance systems, outline methods and provide case studies for assessing malaria surveillance system strengths and identifying gaps, discuss ways to leverage existing services (e.g., community health workers, antenatal care programs, the private sector) for a more robust surveillance system, and highlight the importance of country leadership and ownership for effective and sustainable malaria surveillance. Effectively transforming malaria surveillance into a core intervention requires global leadership and collaboration among national programs; but, more importantly, needs the dedication and empowerment of subnational and local leaders. A fully operational surveillance system that effectively links data quality, data use, and data-to-action components will ultimately lead to a resilient and robust system capable of responding to all malaria transmission contexts, and will facilitate improved malaria control and eventual malaria elimination.

## References

[1] WHO , 2021. *Global Technical Strategy for Malaria 2016–2030, 2021 Update*. Geneva, Switzerland: World Health Organization. Available at: www.who.int/publications/i/item/9789240031357. Accessed February 24, 2022.

[2] Nsubuga P Disease Control Priorities in Developing Countries, 2nd edition. Washington, DC: The International Bank for Reconstruction and Development/The World Bank, Chapter 53. 21250309

[3] WHO , 2015. *Gobal Technical Strategy for Malaria 2016–2030*. Geneva, Switzerland: World Health Organization. Available at: www.who.int/publications/i/item/9789240031357. Accessed February 24, 2022.

[4] Smith R Woodward D Acharya A Beaglehole R Drager N, 2004. Communicable disease control: a ‘global public good’ perspective. Health Policy Plan 19: 271–278. 15310662 10.1093/heapol/czh032

